# Molecular Characterization of *Fasciola* spp. from Some Parts of Iran

**Published:** 2020-01

**Authors:** Hamid HASANPOUR, Reza FALAK, Saied Reza NADDAF, Santiago MAS-COMA, Mohammad Bagher ROKNI, Alireza BADIRZADEH, Kobra MOKHTARIAN, Mehdi MOHEBALI, Sanaz JAFARPOUR AZAMI, Arezoo FADAVI, Mohammad Javad GHARAGOZLOU, Kazem MOHAMMAD, Gholamreza MOWLAVI

**Affiliations:** 1 Department of Parasitology and Mycology, School of Public Health, Tehran University of Medical Sciences, Tehran, Iran; 2 Department of Parasitology, Faculty of Medicine, Ilam University of Medical Sciences, Ilam, Iran; 3 Immunology Research Center, Iran University of Medical Sciences, Tehran, Iran; 4 Department of Parasitology, Pasteur Institute of Iran, Tehran, Iran; 5 Department of Parasitology, Faculty of Pharmacy, University of Valencia, Valencia, Spain; 6 Department of Parasitology and Mycology, School of Medicine, Iran University of Medical Sciences, Tehran, Iran; 7 Department of Parasitology and Mycology, School of Medicine, Shahrekord University of Medical Sciences, Shahrekord, Iran; 8 Department of Pathobiology, School of Veterinary Medicine, University of Tehran, Tehran, Iran

**Keywords:** *Fasciola hepatica*, *Fasciola gigantica*, Iran

## Abstract

**Background:**

Identification of liver flukes, *Fasciola hepatica*, and *Fasciola gigantica* by morphometric parameters is not always reliable due to the overlapping measurements. This study aimed to characterize the liver flukes of animals from different parts of Iran by the genetic markers, ITS1, and *COXI*.

**Methods:**

We collected flukes from infected livestock in six provinces of Iran from Sep to Nov 2016. The flukes were identified by amplification of a 680 bp sequence of ITS1 locus followed by a restriction fragment polymorphism (RFLP) assay. The genetic diversity among isolates was evaluated by amplification and sequencing of a 493 bp fragment of the *COXI* gene.

**Results:**

We obtained 38 specimens from Khuzestan, 22 from Tehran, 10 from Isfahan, 10 from Mazandaran, 4 from Kurdistan, and 3 from Ardabil provinces. PCR-RFLP analysis revealed two patterns, representing *F. hepatica*, and *F. gigantica*. Fifty specimens from cattle and sheep exhibited *F. hepatica* pattern and 37 from the cattle, sheep, buffalo, and goat that of *F. gigantica*. The phylogeny based on *COXI* revealed two distinct clades separating *F. hepatica* from *F. gigantica*. In our phylogeny, the Iranian *F. gigantica* isolates showed a distinct separation from the African flukes, while grouped with the East Asia specimens demonstrating a common ancestor. The *F. hepatica* isolates clustered with the flukes from different parts of the world, including East Asia, Europe, and South America.

**Conclusion:**

The present study revealed a substantial genetic difference between *F. gigantica* populations of Asia and Africa, while *F. hepatica* isolates from different parts of the world shared high similarities.

## Introduction

Fascioliasis is a significant food-borne zoonotic disease worldwide, affecting various mammals, and humans (1). About 180 million of the world population are at the risk of this infection (2), and 2.4 to 17 million or even higher numbers depending upon the hitherto unknown situations in many countries are estimated to be infected (3). In the animal husbandry industry, the economic losses associated with this disease are at around two billion US dollars (1). Two flukes, *Fasciola hepatica,* and *Fasciola gigantica* are responsible for fascioliasis in humans and animals (1, 4, 5) with a higher severity for the latter species due to its bigger size and the greater body mass (6). In Iran, fascioliasis is an endemic disease of herbivores with prevalence ranging from 1.18% to 50% in different geographical regions (7–10). The infection is of the higher rates among animals in the south of the country, while most human cases occur along the Caspian Sea littoral in the north. During 1988–1998, two significant outbreaks struck Gilan Province, infecting ≈15000 people (11, 12). The Caspian Sea littoral has remained a hot spot for the disease. In the west and northwest, in Kermanshah and Ardabil provinces, human fascioliasis appears sporadically with limited outbreaks in the former one (13–15). Moreover, in the areas with high rates of the infection among local livestock, e.g., Lorestan, and Kohgiloye and Boyer-Ahmad, serology detected anti-*Fasciola* antibodies in humans (16, 17). The flukes, *F. hepatica,* and *F. gigantica* are commonly identified based on morphologic and morphometric parameters (11). However, intermediate forms, presumably hybridizations of the two species, are hardly distinguishable by this approach (2). Reports of intermediate forms are available from different Asian countries, including China (18), Korea (19), Japan (20), Vietnam (21), and Iran (11, 22), as well as Egypt in Africa.

Today, various molecular markers, e.g., ITS1, ITS2, 28S rRNA, *COXI*, and *NADI,* are available for molecular identification of *Fasciola* spp. (2, 4, 18). Due to the conserved and variable regions and high copy numbers, ribosomal DNA (rDNA) has proved as a discriminating tool for identification of *Fasciola* species (2), whereas mtDNA sequences with higher mutation rates, lack of recombination and maternal inheritance serve as biomarkers for phylogenetic studies and genetic variability (23). In this study, by using the molecular markers, ITS1, and *COXI*, we characterized the liver flukes of livestock from different regions of Iran.

## Materials and Methods

### Study area

**Fig. 1:**
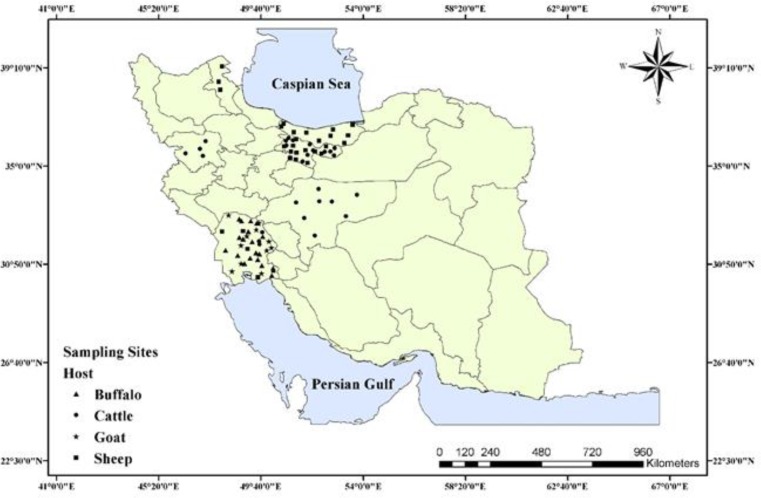
The localities from which *Fasciola* spp. Specimens were obtained

The samples were collected from six provinces of Iran with different geological and weather features including Ardebil in the northwest, Tehran in the north center, Isfahan in the center, Mazandaran in the north, Kurdistan in the west, and Khuzestan in the southwest during Sep to Nov 2016 (Fig. 1). Table 1 shows the climatological features in different regions of the study area.

**Table 1:** Data of *Fasciola* spp. isolates obtained from different regions of Iran and the climate profiles

***Locality***	***Number of samples***	***Host***	***Altitude (m)***	***Average temperature (°C)***	***Precipitation (mm)***		***DNA type (Species)2***
						Climate1	ITS1 COX1
Ardabil	2	Sheep	1351	9.5	325	BSk	F.h F.h
Tehran	4	Cattle	1168	16.4	220	BSk	F.h F.h
Tehran	3	Sheep	1168	16.4	220	BSk	F.h F.h
Isfahan	2	Cattle	1578	15.6	125	BWk	F.h F.h
Mazandaran	4	Sheep	43	16.7	690	Csa	F.h F.h
Khuzestan	2	Goat	19	24.9	345	BWh	F.g F.g
Khuzestan	2	Buffalo	19	24.9	345	BWh	F.g F.g
Kurdistan	3	Cattle	1499	12.8	492	Csa	F.h F.h
Khuzestan	2	Sheep	19	24.9	345	BWh	F.g F.g
Khuzestan	1	Sheep	19	24.9	345	BWh	F.h F.h
Ardabil	1	Sheep	1351	9.5	325	BSk	F.h NP
Mazandaran	6	Sheep	43	16.7	690	Csa	F.h NP
Isfahan	8	Cattle	1578	15.6	125	BWk	F.h NP
Tehran	8	Cattle	1168	16.4	220	BSk	F.h NP
Tehran	7	Sheep	1168	16.4	220	BSk	F.h NP
Kurdistan	1	Cattle	1499	12.8	492	Csa	F.h NP
Khuzestan	1	Sheep	19	24.9	345	BWh	F.g NP
Khuzestan	9	Goat	19	24.9	345	BWh	F.g NP
Khuzestan	3	Cattle	19	24.9	345	BWh	F.g NP
Khuzestan	18	Buffalo	19	24.9	345	BWh	F.g NP

1 BSk, Cold semi-arid climates; Csa, Mediterranean climate; BWh, Desert climate

2 Fh, *Fasciola hepatica*; Fg, *Fasciola gigantica*; NP, Not performed

### Sample collection

We obtained 87 *Fasciola* flukes from different infected livestock, including sheep (n=29), goat (n=11), cattle (n=27), and Buffalo (n=20) slaughtered in the six provinces. The flukes were transferred to the Laboratory of Helminthology, School of Public Health, Tehran University of Medical Sciences, extensively washed with PBS and preserved in 70% alcohol, and kept at room temperature until used.

### DNA extraction

Genomic DNA was extracted from a portion of the apical zone of the flukes. The tissue was ground using a surgical blade, and DNA extraction was performed by a commercial DNA extraction kit (Bioneer Corporation, Daejeon, South Korea) according to the manufacturer's instructions. The extracted DNAs were stored at −20 °C until used.

### ITS1-PCR and RFLP analysis

A 680 bp fragment of ITS1 locus was targeted by using the primers (Table 2) designed by others (19) and synthesized in a commercial company (Macrogen Corporation, Seoul, South Korea). The 25 μl reactions contained 1 μl of the template DNA, 10 μl of master mix (0.2 U *Taq* DNA polymerase, 2 mM MgCl_2_, 400 pM dNTPs and the buffer system (Ampliqon, Skovlunde, Denmark), 200 pM each of forward and reverse primer, and double-distilled water (DDW) to the final volume. The PCR amplification programmed for an initial denaturation of 10 min at 94 °C followed by 25 cycles of 94 °C for 90 sec, 58 °C for 90 sec, and 72 °C for 90 sec with a final extension of 10 min at 72 °C. Amounts of 3 μl from amplicons were run on 1.5% gels at 90 V for 90 min, stained with 2% DNA safe stain® (Pishgam Biotech Co., Tehran, Iran) and visualized under UV (Syngene, Cambridge, UK). In all amplifications, DNA from a previously identified *F. hepatica* fluke (24) and DDW were included as positive and negative controls, respectively.

**Table 2:** Primers used for amplification of ITS1 and COXI fragments in this study

***Target gene***	***Primers***	***Sequence (5′-3′)***	***expected band (bp)***	***Reference***
ITS1	ITS1-Forward	TTGCGCTGATTACGTCCCTG	680	(19)
	ITS1-Reverse	TTGGCTGCGCTCTTCATCGAC		
COX1	Ita8-Forward	ACGTTGGATCATAAGCGTGT	493	(20)
	Ita9-Reverse	CCTCATCCAACATAACCTCT		

ITS1, Internal transcribed spacer 1; COX1, Cytochrome oxidase subunit I

Identification of *F. hepatica* and *F. gigantica* species was performed by a restriction fragment polymorphism (RFLP) assay using the *RsaI* enzyme (Fermentas, Waltham, United States) as described elsewhere (25)*.* The reactions contained 5 μl of PCR product, 5 μl of the enzyme, 2 μl of the buffer, and DDW to a final volume of 22 μl. The mixture incubated overnight at 37 °C followed by electrophoresis on 2% agarose gels and staining with 2% DNA safe stain. The ITS1 types were identified according to the generated patterns reported in other studies (25).

### COXI amplification and phylogenetic analysis

The genetic diversity among *Fasciola* species was evaluated by amplification of a 493 bp sequence of *COX*I (20) of *F. hepatica* (n=19), and *F. gigantica* (n=6) flukes obtained from different animals including cattle, sheep, buffalo, and the goat (Table 1). The 25 μl reactions contained, 10 μl of master mix (0.2 U *Taq* DNA polymerase, 2 mM MgCl_2_, 400 pM of each dNTPs and buffer system) (Ampliqon, Skovlunde, Denmark), 200 pM each of forward and reverse primer, 1 μl DNA template, and DDW to the final volume. The PCR amplification program included an initial denaturation of 10 min at 94 °C followed by 25 cycles of 94 °C for 90 sec, 56 °C for 90 sec, and 72 °C for 90 sec with a final extension of 10 min at 72 °C. The amplicons were sequenced in the forward direction using the same primer used for amplification by a 23 ABI 3730XLs sequencer. (Macrogen Corporation, Seoul, South Korea).

### Blast analysis

The generated sequences were manually corrected and compared with similar sequences of the *F. hepatica* and *F. gigantica* available in GenBank database by the Basic Local Alignment Search Tool (BLAST) program (https://blast.ncbi.nlm.nih.gov/Blast.cgi?PAGE_TYPE=BlastSearch)

### Phylogeny

The *COXI* sequences generated in this study were aligned with similar sequences belonging to various *Fasciola* spp. isolates from Iran and other countries, including those representing the intermediate form. The distance between the sequences was calculated, and a phylogenetic tree was constructed by using the Jukes-Cantor option of the neighbor-joining method in a pairwise deletion procedure using MEGA 7 software (26). The robustness of the topologies was estimated through 1,000 bootstrap replications.

### Geographical analysis

ArcGIS 10.2 GIS software was used to draw maps of Iran and show the weather conditions, altitude, and average temperature.

## Results

### ITS1-PCR and RFLP analysis

**Fig. 2:**
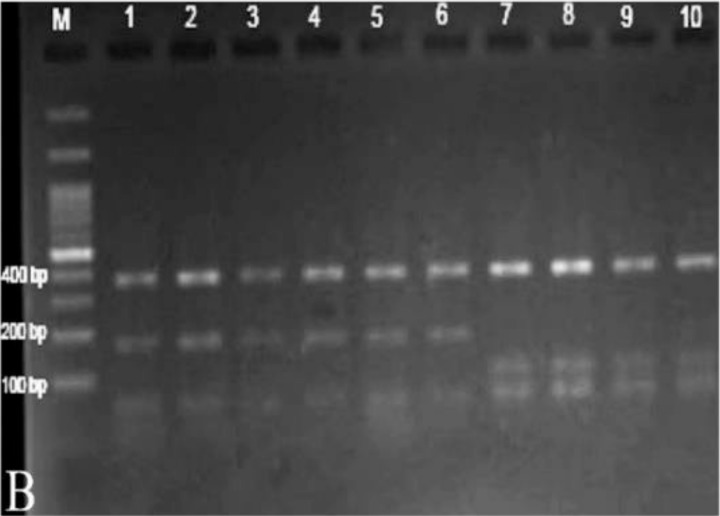
RFLP analysis of ITS1-PCR products using *RsaI*. Lane M, a 100-bp size marker (Jena Bioscience, Jena, Germany); lanes 1 to 6, *F. gigantica*; lanes 7 to 10 *F. hepatica*

In PCR amplification, all the specimens and positive controls yielded the expected ≈680 bp amplicon. In RFLP analysis, the digestion generated two patterns: one comprised three bands of approximately 60 bp, 170 bp and 370 bp representing *F. gigantica*, and the other three bands of 60 bp, 100 bp, and 370 bp indicating *F. hepatica* (25). Of the 87 specimens, 50 (57.4%) from the cattle and sheep revealed the *F. hepatica* pattern, and 37 (42.6%) from the cattle, sheep, buffalo, and goat showed that of *F. gigantica*. No intermediate pattern was detected by this approach (Table 1 and Fig. 2).

### BLAST analysis

In BLAST analysis, the *F. hepatica COXI* sequences generated here showed 98%–99% identity (97%–100% coverage) with similar sequences from the center (Acc. No. KU946983), northwest (Acc. No. KX021278) and northeast (Acc. No. KX021290) of Iran and other countries, *e.g.*, Poland (Acc. No. KR422380), and Australia (Acc. No. AF216697). The *F. gigantica COXI* sequences exhibited 98%–99% similarity with the isolates from the southwest (Acc. No.Q398050), southeast (Acc. No. KX036349), center (Acc. No. KX712305) and northwest (Acc. No. KX063835.1) of Iran. The sequences had a 98% similarity over 98%–100% coverage with the isolates from China (Acc. No. KF543343), India (Acc. No. KX656877), Vietnam (Acc. No. MF287791).

### Similarity and Phylogenetic analysis

The intraspecies variation (within-group mean distance) among *F. hepatica* and *F. gigantica COXI* sequences were 3.44% and 11.99%, respectively. Much of the intraspecies variation in the *F. gigantica* cluster was due to Zambia sequences showing a considerable distance from the rest of Africa (20.10%) and other parts of the world (≈22.2%). The variations among Iranian *F. hepatica* and *F. gigantica COXI* sequences generated herein were 2.74% and 5.16%, respectively.

**Fig. 3:**
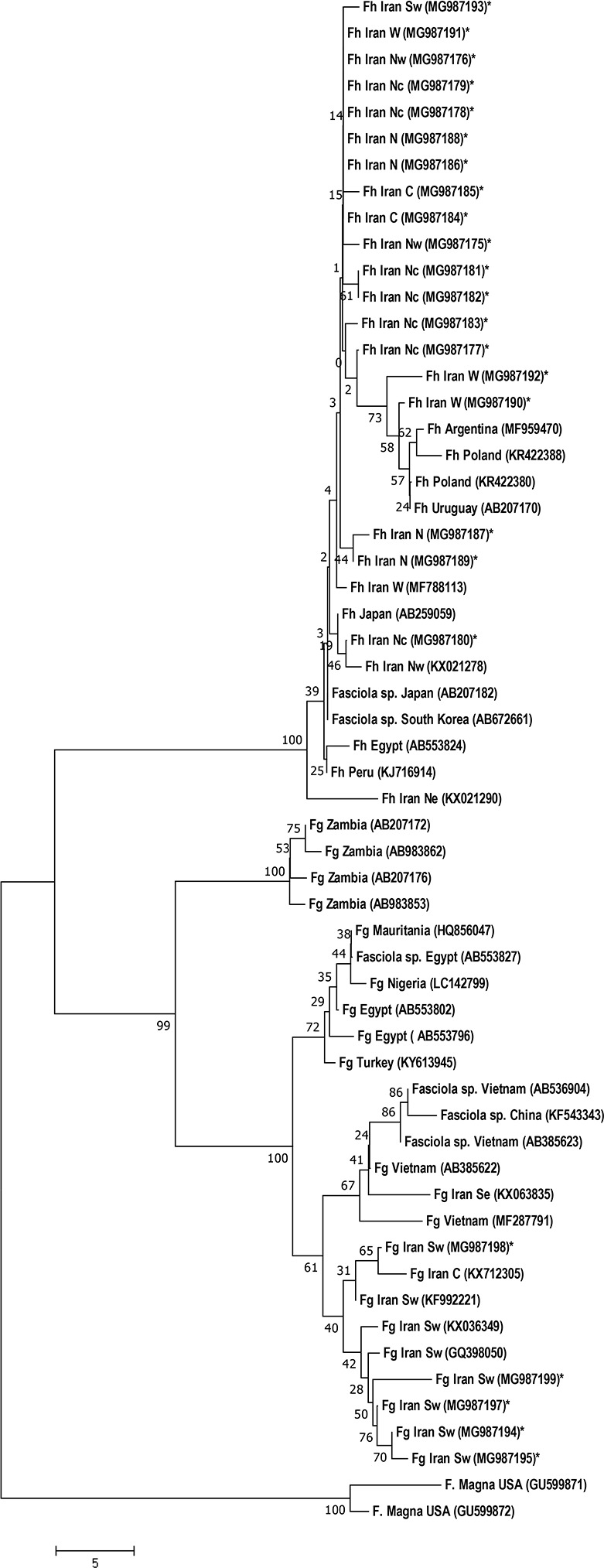
Phylogeny of *Fasciola* sp. based on the *COXI* gene constructed by Neighbor-joining method with the Jukes-Cantor option of the neighbor-joining method in a pairwise deletion procedure using MEGA 7 software. The scale bar corresponds to a 5% distance. The accession numbers of sequences used for the construction of the tree are shown in parentheses and the sequences generated in this study by an asterisk

In phylogeny, the *COXI* sequences clustered in two groups with distinct separation of two species. In the *F. gigantica* group, the sequences from Zambia grouped in a clade separate from other sequences. Besides, the sequences of African countries, i.e., Mauritania, Nigeria, and Egypt, and a sequence from Turkey grouped close together distinct from those of other countries, including Iran. The intermediate forms of Vietnam and China made a separate cluster close to other sequences from Vietnam and a sequence from Iran, while the intermediate from Egypt grouped with sequences from Mauritania and Nigeria, and close to other *F. gigantica* sequences from Egypt. In the *F. hepatica* group, the sequences from Europe (Poland) and two sequences of South America (Argentina and Uruguay) clustered close near two sequences obtained in this study. Moreover, the two Japanese and South Korea intermediated forms grouped close to *F. hepatica* sequences from Peru, Japan, and Iran (Fig. 3). Different phylogenetic approaches demonstrated almost the same topology.

## Discussion

**Fig. 4:**
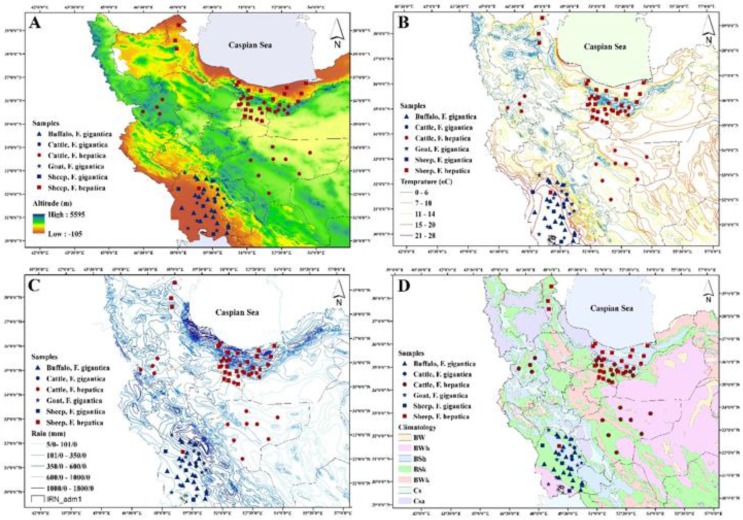
Distribution of *Fasciola* spp. in sampling localities and their relevant hosts according to A) altitude B) temperature C) precipitation and D) climate

We investigated the presence of *Fasciola* species in some parts of Iran using the molecular markers ITS1 and *COXI*. Many reports on the identification and distribution of the *Fasciola* spp. are available from Iran. In the absence of molecular analysis, a morphometric comparison of the specimens from Gilan Province with the standard allopatric populations of *F. hepatica* and *F. gigantica* revealed two distinct types with some overlapping specific measurements indicating the intermediate form (11). Later, ITS sequencing of specimens from the neighboring province, Mazandaran, identified three genotypes, including *F. hepatica*, *F. gigantica*, and the intermediate forms, while no agreement between morphometric and molecular analysis was demonstrated (22). In Zanjan Province, Midwest of the Iran, morphometry identified both species and the intermediate forms, while ITS2 revealed only one genotype representing *F. hepatica* (27). In eastern Iran, ITS2-RFLP and *ND1* sequencing elucidated the occurrence of *F. gigantica* in southern regions, while at the upper latitudes, most cattle harbored both species (28). In Khuzestan Province, southwest of the country, the 28S marker revealed *F. gigantica* and *F. hepatica,* with the former one as the dominant species (29). Our present study identified *F. hepatica* in Ardebil, Tehran, Isfahan, Mazandaran, and Kurdistan, and *F. gigantica* in Khuzestan, as the primary species (Fig. 1). These data are in agreement with the previous works exhibiting *F. gigantica* as the dominant species in the south, southwest, and southwest of Iran and *F. hepatica* as the more widespread species in north and northwest of Iran (9, 22, 30). In areas with a temperature gradient resulting from various altitudes, the two species exhibit distinct distributions. In Gilan Province, northern Iran, *F. hepatica* commonly occurs in the highlands while *F. gigantica* is more prevalent in the herbivores of the lowlands (11). We obtained *F. hepatica* flukes from areas with a mean elevation of 1127±80 m above sea level. In Khuzestan Province, a subtropical region with 19±00 m elevation, *F. gigantica,* was the dominant species, and *F. hepatica* was detected only in one sheep (Table 1, Fig. 4).

Our phylogeny clustered the specimens into two distinct clades. The intermediate forms (*Fasciola* sp.) grouped with either *F. hepatica* or *F. gigantica* clade demonstrating the difference in maternal mtDNA. The emergence of these forms, presumably hybrids of two species in different geographical areas is a matter of controversy. In Egypt, mixed infections of spermic species in animals indicate cross-hybridization of the two species as reflected by codominant inheritance of ITS alleles (4), while in East Asian countries like Vietnam, South Korea, Japan, and China, intermediate forms appear as aspermic usually triploid flukes with parthenogenetic reproduction (31). In our phylogeny, the Iranian *F. gigantica* isolates showed a distinct separation from the African fluke and grouped with the East Asia specimens demonstrating a common ancestor. Our *F. hepatica* isolates clustered with the isolates from different parts of the world, including East Asia, Europe, and South America (Fig. 3). In our phylogenetic tree, a fluke from Japan previously reported a heterozygote with *F. gigantica* mtDNA background (20) grouped with *F. hepatica* clade reflecting the genuine maternal inheritance of this specimen (Fig. 3). The PCR-PFLP has shown a reliable and precise method for the detection of *Fasciola* species (25). In this study, PCR-PFLP detected no overlapping pattern indicating the intermediate forms. One flaw in our study was the lack of specimens from Gilan Province, where morphometry previously identified the intermediate form.

Further studies with specimens from areas where two species co-occur (11) might reveal with more precision the molecular identity of *Fasciola* species in Iran and the possible occurrence of the intermediate form. Our study revealed *F. hepatica* as the primary cause of animal fascioliasis in Ardebil, Tehran, Isfahan, Mazandaran, and Kurdistan provinces, and *F. gigantica* as the common species in Khuzestan Province.

## Conclusion

The present study revealed a substantial genetic difference between *F. gigantica* populations of Asia and Africa, and high genetic similarities between *F. hepatica* isolates from different parts of the world.

## Ethical considerations

Ethical issues (including plagiarism, informed consent, misconduct, data fabrication and/or falsification, double publication and/or submission, redundancy, etc.) have been completely observed by the authors.
